# MicroRNAs in the miR-17 and miR-15 families are downregulated in chronic kidney disease with hypertension

**DOI:** 10.1371/journal.pone.0176734

**Published:** 2017-08-03

**Authors:** Priyanka Nandakumar, Adrienne Tin, Megan L. Grove, Jianzhong Ma, Eric Boerwinkle, Josef Coresh, Aravinda Chakravarti

**Affiliations:** 1 McKusick - Nathans Institute of Genetic Medicine, Johns Hopkins School of Medicine, Baltimore, Maryland, United States of America; 2 Predoctoral Training Program in Human Genetics and Molecular Biology, McKusick-Nathans Institute of Genetic Medicine, Johns Hopkins University School of Medicine, Baltimore, Maryland, United States of America; 3 Johns Hopkins Bloomberg School of Public Health, Welch Center for Prevention, Epidemiology, and Clinical Research, Baltimore, Maryland, United States of America; 4 Human Genetics Center, Department of Epidemiology, Human Genetics and Environmental Sciences, School of Public Health, The University of Texas Health Science Center at Houston, Houston, Texas, United States of America; 5 Human Genome Sequencing Center, Baylor College of Medicine, Houston, Texas, United States of America; University Medicine Greifswald, GERMANY

## Abstract

**Background:**

In older adults (aged 70–74 years), African-Americans have 4-fold higher risk of developing hypertension-attributed end-stage renal disease (ESRD) than European-Americans. A hypothesized mechanism linking hypertension and progressive chronic kidney disease (CKD) is the innate immune response and inflammation. Persons with CKD are also more susceptible to infection. Gene expression in peripheral blood can provide a view of the innate immune activation profile. We aimed to identify differentially expressed genes, microRNAs, and pathways in peripheral blood between cases with CKD and high blood pressure under hypertension treatment versus controls without CKD and with controlled blood pressure in African Americans.

**Methods:**

Case and control pairs (N = 15x2) were selected from those without diabetes and were matched for age, sex, body mass index, *APOL1* risk allele count, and hypertension medication use. High blood pressure under hypertension treatment was defined as hypertension medication use and systolic blood pressure (SBP) ≥ 145 mmHg. CKD was defined as estimated glomerular filtration rate (eGFR) < 60 mL/min/1.73m^2^. Cases were selected from those with CKD and high blood pressure under hypertension treatment, and controls were selected from those without CKD (eGFR: 75–120 mL/min/1.73m^2^ and urine albumin-to-creatinine ratio < 30mg/g) and with blood pressure controlled by hypertension medication use (SBP < 135 mmHg and D(diastolic)BP < 90 mm Hg). We perform RNA sequencing of mRNA and microRNA and conducted differential expression and co-expression network analysis.

**Results:**

Of 347 miRNAs included in the analysis, 14 were significantly associated with case status (Benjamini-Hochberg adjusted p-value [BH p] < 0.05). Of these, ten were downregulated in cases: three of each belong to the miR-17 and miR-15 families. In co-expression network analysis of miRNA, one module, which included 13 of the 14 significant miRNAs, had significant association with case status. Of the 14,488 genes and 41,739 transcripts included in the analysis, none had significant association with case status. Gene co-expression network analyses did not yield any significant associations for mRNA.

**Conclusion:**

We have identified 14 differentially expressed miRNAs in the peripheral blood of CKD cases with high blood pressure under hypertension treatment as compared to appropriate controls. Most of the significant miRNAs were downregulated and have critical roles in immune cell functions. Future studies are needed to replicate our findings and determine whether the downregulation of these miRNAs in immune cells may influence CKD progression or complications.

## Introduction

About one-third of end-stage renal disease (ESRD) in the U.S. has been attributed to hypertension [1]. In older adults, African-Americans have 4-fold higher risk of developing hypertension-attributed ESRD than European-Americans, and the incidence of hypertension-attributed ESRD increases with age [2]. A hypothesized mechanism linking hypertension and kidney function decline is the innate immune response and inflammation [3]. Inflammation biomarkers in blood have been associated with kidney function decline and incident hypertension [4–7]. Additionally, older adults with chronic kidney disease (CKD) have increased risk of infection-related hospitalization or bloodstream infection [8,9]. Although immune dysfunction in persons with ESRD due to metabolic disorder and retention of uremic solute is well established [10,11], the mechanisms underlying the links between immune function, hypertension, and kidney function in earlier stages of CKD is not well understood. Gene expression analysis in peripheral blood can provide a view of the innate immune activation profile and may lead to insights into the pathophysiology of hypertension and CKD, and their complications.

Studies using immune cells have identified specific gene expression profiles of CKD patients versus hemodialyzed patients, and of patients with essential hypertension versus controls [12,13]. Recently microRNAs (miRNA) have been shown to have a potential role in influencing blood pressure and kidney function through modulating the immune response [14–16]. miRNAs are small noncoding RNAs (~22 nucleotides in length) with important post-transcriptional regulatory functions and are an attractive target of investigations.

Given that African Americans have a strong predisposition for hypertension-attributed kidney disease, we decided to study hypertension and kidney disease jointly in older African Americans. We aimed to identify differentially expressed genes and miRNAs in the peripheral blood to gain insights into the immune profile of hypertension and kidney disease. We used a matched case-control design (n = 15x2) in the African Americans cohort of the Atherosclerosis Risk in Communities (ARIC) study [17]. We conducted RNA sequencing to quantify expression levels and performed differential expression and co-expression network analyses to identify gene expression profiles in the peripheral blood of hypertension and CKD cases.

## Methods

### Ethics statement

Written informed consent was obtained from all ARIC study participants, and approval was granted from the relevant institutional review boards (IRB) for the participating study centers (University of North Carolina, University of Minnesota, University of Mississippi Medical Center, and Johns Hopkins University). JHSPH IRB No. H.34.99.07.02.A1, study title “Atherosclerosis Risk in Communities (ARIC) Study—Morbidity/Mortality Follow-up Field Center.” This research was conducted in accordance with the principles described in the Declaration of Helsinki.

### Study design

The ARIC Study is an ongoing prospective cohort study in four US communities [17]. A total of 15,792 participants aged 45–64 years were recruited from Forsyth County, North Carolina; Jackson, Mississippi; suburban Minneapolis, Minnesota; and Washington County, Maryland between 1987 and 1989 (Visit 1). Four follow-up examinations (visits 2–5) have been conducted. Blood samples preserved for RNA analysis using PAXgene blood tubes were available from visit 5 (2011–13).

Case and control pairs (N = 15 pairs) were selected from those individuals without diabetes but on hypertension treatment and they were matched by age, sex, body mass index, *APOL1* renal risk allele count, and hypertension medications (ACE inhibitor, angiotensin receptor blocker, and calcium channel blocker) to reduce heterogeneity. The use of hypertension medication was included in the selection criteria because a high proportion (>60%) of older African Americans were on hypertension treatment [18], and some hypertension medications have been reported to influence gene expression in peripheral blood [19]. In the ARIC study, hypertension medication use was determined by inspection of medication bottles at study visit. High blood pressure under hypertension treatment was defined as systolic blood pressure (SBP) ≥ 145 mmHg with hypertension medication use. CKD was defined as estimated glomerular filtration rate (eGFR) < 60 mL/min/1.73m^2^. Cases were selected from those with CKD and high blood pressure under hypertension treatment. Controls were selected from those with blood pressure controlled by hypertensive medications (SBP < 135 mmHg and D(diastolic)BP < 90 mm Hg) and without CKD (eGFR: 75–120 mL/min/1.73m2 and urine albumin-to-creatinine ratio [UACR] < 30mg/g). The *APOL1* renal risk variants are strongly associated with CKD progression [20], thus matching by *APOL1* risk variants provided the opportunity to identify differential expressed genes that were independent of *APOL1*. The characteristics of the cases and controls were compared using the t-test, Kruskal-Wallis, or Fisher’s exact test, as appropriate.

*mRNA and miRNA library preparation*, *sequencing*, *processing*, *alignment*, *and quantitation methods* are described in S1 Text.

### Surrogate variable generation

Surrogate variables (SVs) representing unknown confounders were estimated using the svaseq function from the R package sva [21], with the null model absent of variables, and case-status as the variable of interest in the full model. SVs were included as covariates in models in all subsequent expression analyses as expanded upon in the Differential Expression Analyses Methods sections.

### mRNA differential expression analyses

The mRNA analyses were conducted on genes and transcripts from a list of 20,377 genes tagged as “protein-coding” in the GENCODE V19 annotation files. Results were adjusted within each analysis using the Benjamini-Hochberg [22] procedure for multiple testing.

The R package DESeq2 [23] was used to conduct gene-level analyses, with the null hypothesis of a zero log2-fold change. As DESeq2 incorporates normalization and default outlier replacement procedures, SVs were generated on DESeq2 outlier-replaced, normalized counts and included as covariates for these analyses. Gene-level analyses were carried out on14,488 genes with a normalized count ≥1 in at least 14 out of 29 samples (~50% as one sample failed library preparation, see Results), with a target FDR of alpha = 0.05 used for independent filtering. SVs were generated using 10,524 genes with a minimum normalized count of 10 in all 29 samples.

Gene-level kidney-focused analyses were also conducted, in which 397 kidney-expressed proteins from the Human Protein Atlas [24] (v14) (http://www.proteinatlas.org/humanproteome/kidney) were examined. Of these, 392 were available in our data; four were not available in the ENSEMBL GRCh37 assembly, and one was not in our final GTF of protein-coding genes. Genes meeting the normalized count threshold defined as above for expression were subset to produce the final kidney-specific results.

The Ballgown [25] R package was used to analyze 41,739 transcripts with fragments per kilobase of transcript per million mapped reads (FPKM) ≥ 0.3 in at least 14 samples, and SVs were generated on transcripts with FPKM ≥ 0.5 in at least 26 samples (90%) (17,085 transcripts).

### miRNA differential expression analyses

The miRNA analyses were conducted on mature miRNAs from miRBase [26] v20 and novel miRNAs, detected as described in S1 Text. Similar to the mRNA analyses, the R package DESeq2 was used for miRNA analyses with the null hypothesis of a zero log2-fold change, and SVs were generated on the outlier-replaced normalized counts and included as covariates in the model. These analyses were carried out on 347 miRNAs with a normalized count ≥ 1 in at least 15 out of 30 samples (50%) with target FDR of alpha = 0.05 used for independent filtering, and SVs were generated using 270 miRNAs with a minimum normalized count of 3 in at least 27 samples (90%). Counts were summed across all precursors for each mature miRNA. Results were adjusted using the Benjamini-Hochberg procedure for multiple testing. We performed hierarchical clustering of the normalized counts of the miRNAs with nominal p-value < 0.01 to visualize the expression levels of these miRNAs between cases and controls.

### Co-expression network analysis

We used the Weighted Gene Co-Expression Network Analysis (WGCNA) R package to construct correlated signed network and tested for associations between the eigengene (the first principal component) of each module and case status [27]. The genes, transcripts, and miRNAs used for co-expression network analysis and the generation of surrogate variables was the same as those for differential expression analyses. The gene and miRNA counts were first normalized by the size factor with outlier replacement using DESeq2 [23]. The transcript counts were normalized to FPKM using Ballgown [25]. Next, the gene, transcript, or miRNA expression levels were transformed using natural logs after adding 1. Residuals of the log transformed expression levels were generated with adjustment for surrogate variables. These residuals were used as input for co-expression analysis. In network construction, we used bi-weight mid-correlation as a measure of co-expression to minimize the influence from outliers. For soft thresholding power, we used the first power with adjusted R square for linear fit > 0.8. The minimum module size was set as 25, 30, and 8 for genes, transcripts, and miRNA, respectively. The significant threshold for module association was defined as 0.05 divided by the number of correlated modules in each specific analysis.

### miRNA target prediction and gene ontology analyses

The procedure for miRNA target prediction and gene ontology analyses of differentially expressed miRNAs is similar to the procedure described by the authors of the empiricalGO [28] software in Bleazard et al. 2015. Briefly, miRanda [29] v3.3a was used for miRNA target prediction with parameters free energy < −20 kcal/mol and score > 155 [28]. The 3’UTR sequences for target prediction were obtained from GRCh37 ENSEMBL BioMart [30]. Subsequently, gene ontology (GO) annotations were obtained from GRCh37 ENSEMBL BioMart, and the empiricalGO python script was used in “basic” mode to produce a list of GO terms with a one-tailed permutated p-value for each term. The final results were subset to include only terms with a minimum size of 10, and the Benjamini-Hochberg multiple testing correction was applied to adjust for the number of GO terms in each list.

## Results

### Study population characteristics

The mean age of the cases and controls was 77 years, and 67% were female. No significant differences were detected between cases and controls for all of the matching characteristics (Table 1). The cases had significantly lower eGFR (mean of 46 min/mL/1.73m^2^ vs 88 min/mL/1.73m^2^, p < 0.0001) and higher SBP (mean of 156 mm Hg vs. 115 mm Hg, p < 0.0001). UACR was higher in cases (median of 13.7mg/g vs 7.8mg/g), but the difference was not significant (p = 0.13). On hypertension medications, none of the participants were on both angiotensin-converting-enzyme (ACE) inhibitor and angiotensin receptor blocker while two participants were on ACE inhibitor and beta blocker, and four participants were on ACE inhibitor and calcium channel blocker. These participants were split evenly between the case and control groups. Since gene expression levels were measured in whole blood, we also compared cases and controls for white blood cell count and percentage of lymphocyte, monocyte, and granulocyte, and did not observe significant differences.

**Table 1:** Study population characteristics.

Variable	Case	Control	p
**Selection variables**			
eGFR, mL/min/m^2^, mean (SD)	46 (12)	88 (10)	N/A
SBP, mm Hg, mean (SD)	156 (12)	115 (33)	N/A
**Matching variables**			
Age, year, mean (SD)	77 (4.5)	77 (5.4)	0.97
Female, % (n)	67 (10)	67 (10)	1.00
BMI, kg/m^2^, mean (SD)	28 (3.5)	30 (3.3)	0.09
APOL1 high-risk, % (n)	33 (5)	33 (5)	1.00
Hypertension medications			
ACE inhibitor, % (n)	53.3 (8)	60 (9)	1.00
Angiotensin receptor blocker, % (n)	27 (4)	27 (4)	1.00
Beta blocker, % (n)	33.3 (5)	33.3 (5)	1.00
Calcium channel blocker, % (n)	47 (7)	60 (9)	0.71
**Blood cell type variables**			
White blood cell count, 1000 per mm^3,^ median (1st, 3rd quartile)	5.4 (4.1, 6.2)	4.8 (4.4, 5.9)	0.80
Lymphocyte, %, median (1st, 3rd quartile)	33.6 (29.1, 39.0)	30.4 (27.8, 40)	0.72
Monocyte, %, median (1st, 3rd quartile)	13.8 (12.8, 17.2)	13.4 (12.5, 16.0)	0.48
Granulocyte, %, median (1st, 3rd quartile)	50.9 (45.1, 57.4)	54.8 (46.3, 59.8)	0.48
**Other variables**			
DBP, mm Hg, mean (SD)	76 (12)	70 (19)	0.31
Albuminuria, median (1st, 3rd quartile)	13.7 (5.9, 66.7)	7.8 (5.7, 13.3)	0.13
High sensitive C-reactive Protein,	1.4 (1.01, 6.87)	5.3 (2.5, 6.3)	0.31
Serum creatinine, mg/dL	1.6 (0.9)	0.8 (0.1)	0.003
Diuretic use, % (n)	73.3 (11)	64.3 (9)	0.70

p, p-value, N/A, not applicable

### Sequencing and processing of mRNAs and miRNAs

Sequencing of mRNA was successful for 29 out of 30 samples; one sample (case) failed library preparation. All 30 samples were successfully sequenced for miRNA. Quality control (QC) and mapping statistics for the 29 samples with mRNA data (depth: 18.7M-45.1M paired-end reads) and the 30 samples with miRNA data (depth: 6.2M-11.5M single-end reads) are listed in S1 and S2 Tables, respectively. As S1 Table indicates, External RNA Controls Consortium (ERCC) transcripts were poorly detected in one sample, but this individual was retained for analysis because the distribution of counts in other genomic features aligned with those of the other samples, indicating a possible issue with the ERCC spike-in itself.

### Differential expression analysis of genes, transcripts, and miRNAs

The gene-level differential expression analysis of 14,448 protein-coding genes considered “expressed” (normalized count ≥ 1 in ≥50% of samples) produced no significant genome-wide results after Benjamini-Hochberg (BH) multiple testing correction (S3 Table). The relevance of the kidney to both hypertension and CKD led us to examine a set of 154 genes meeting our expression threshold and expressed in the kidney, based on data available from the Human Protein Atlas [24], which contains protein expression data from four kidney samples, with proteins in the glomeruli, proximal tubules, distal tubules and the collecting ducts. In this subset of genes, none were significant after BH correction. The gene with the lowest p-value was *SMIM24* (unadjusted p = 5.67x10^-4^, BH p = 0.087).

Transcript-level analyses were conducted on 41,739 transcripts with FPKM ≥ 0.3 in ≥ 50% of samples. After BH correction, there were no differentially expressed transcripts identified; results with p < 10^−3^ are presented in S4 Table.

We tested 347 miRNAs, including 12 novel miRNAs, for differential expression. We detected 14 significant miRNAs. Four were upregulated, and 10 were downregulated in the cases as compared to the controls (Table 2). The most significant miRNA was miR-17-5p (log2 fold change = -0.77, BH p = 6.7E-4). Two other miRNAs in the miR-17 family (miR-106a-5p, miR-106b-3p) were also significantly downregulated. The miR-15 family has three members that were significantly downregulated in the cases (miR-15a-5p, miR-15b-5p, and miR-16-5p). Twenty members of the miR-17 and miR-15 families were found in our data and all, but two, were downregulated in cases, although the associations of some were not significant (S5 Table). S1 Fig presents a heat map of the normalized count of the 32 miRNAs with nominal p-value < 0.01.

**Table 2:** Differentially expressed miRNAs (BH p<0.05).

miRNA	Mean of normalized count	Log2 Fold Change	SE	p	BH p
hsa-miR-17-5p	71.97	-0.77	0.16	1.94x10^-6^	6.76x10^-4^
hsa-miR-130a-3p	492.19	-0.60	0.15	4.31x10^-5^	7.47x10^-3^
hsa-miR-15b-5p	215.32	-0.72	0.19	1.24x10^-4^	1.17x10^-2^
hsa-miR-106b-3p	1425.98	-0.65	0.17	1.35x10^-4^	1.17x10^-2^
hsa-miR-106a-5p	5.46	-1.03	0.28	2.12x10^-4^	1.34x10^-2^
hsa-miR-16-5p	6983.54	-0.64	0.17	2.33x10^-4^	1.34x10^-2^
hsa-miR-181a-5p	15301.70	-0.59	0.16	2.80x10^-4^	1.39x10^-2^
hsa-miR-1285-3p	561.01	-0.44	0.13	4.92x10^-4^	2.14x10^-2^
hsa-miR-15a-5p	430.52	-0.76	0.23	8.36x10^-4^	3.18x10^-2^
hsa-miR-29c-5p	73.85	0.55	0.17	9.18x10^-4^	3.18x10^-2^
hsa-miR-345-5p	715.07	0.60	0.18	1.04x10^-3^	3.27x10^-2^
hsa-miR-142-3p	89.46	0.65	0.20	1.23x10^-3^	3.52x10^-2^
hsa-miR-339-3p	507.26	0.36	0.11	1.32x10^-3^	3.52x10^-2^
hsa-miR-210-3p	440.49	-0.39	0.12	1.50x10^-3^	3.72x10^-2^

SE, standard error; p, p-value; BH p, Benjamini-Hochberg adjusted p-value

Experimentally validated targets in humans were available for 11 of the 14 significant miRNAs in miRTarBase [31] 6.0 (S6 Table). The number of validated target genes for each significant miRNA ranged from 4 (miR-1285-3p) to 134 (miR-17-5p), with a median of 56. Altogether 272 unique genes have experimental evidence of being regulated by the 11 significant miRNAs. Of these, 239 were detected in our data, and 8 were associated with case status at p < 0.05 (S7 Table).

### Co-expression network analysis of genes and transcripts

To investigate whether patterns in gene co-expression may be related to case-status, we conducted gene co-expression analysis. Of the 14,488 genes included in the analysis, 178 were assigned into four co-expression modules (S8 Table). The rest were pruned by WGCNA due to low correlation (< 0.3) with the eigengene in each module. The association between the eigengene of the brown module (consisting of 35 genes) and case status was nominally significant (p = 0.03, S9 Table). Of the 41,739 transcripts included in the analysis, no correlated modules were detected.

### Co-expression network analysis of miRNAs

Of the 347 miRNA included in co-expression analysis, 182 were assigned into five co-expression modules with correlated miRNAs (S10 Table). The rest (n = 165) were pruned due to low correlation with the eigengene in each module. The eigengene of the turquoise module (108 miRNAs) was significantly associated with case status (p = 0.005, Table 3). The eigengene of the blue module (29 miRNAs) was nominally associated with case status (p = 0.03). For both modules, case status was associated with lower eigengene values suggesting an association with lower miRNA expression in these modules. The turquoise module included 13 of the 14 miRNAs that were significantly downregulated in the cases (Table 2).

**Table 3:** Association between the eigengene of each miRNA co-expression module and case status.

Module	Beta^b^	SE	p^a^	Number of miRNAs
Turquoise^c^	-0.97	0.32	5.38x10^-3^	108
Blue	-0.80	0.34	2.63x10^-2^	29
Green	-0.06	0.37	8.63x10^-1^	11
Brown	0.03	0.37	9.32x10^-1^	23
Yellow	-0.01	0.37	9.84x10^-1^	11

SE, standard error; p, p-value

^a^ p-values represent association of the module eigengenes, the first principal component of expression levels of a module, with case status.

^b^ Beta values are in log expression level units.

^c^ 13 out of 14 differentially expressed miRNAs (excluding hsa-miR-339-3p), belong to the turquoise module.

### Gene ontology analysis of significant miRNAs

We then proceeded to predict target genes of the significant miRNAs using miRanda. The predicted targets were analyzed for enrichment of gene ontology annotations using empiricalGO with all predicted targets of the 347 expressed miRNAs as the “universe.” empiricalGO counted 5,576 targets for the 14 differentially expressed miRNAs from the target genes, of which 4,431 had associated GO terms. No GO terms with minimum size of 10 genes in our data were significant after Benjamini-Hochberg multiple test correction, while 25 terms had a one-tailed permutated p < 0.05 (S11 Table). All results for mRNA and miRNA analyses are summarized in S2 Fig.

## Discussion

### Main findings

We have identified 14 miRNAs that were significantly associated with CKD and high blood pressure under hypertension treatment. Ten of these miRNAs were downregulated in the cases, and 13 were grouped in one module in co-expression network analysis. Three of the 14 miRNAs belong to the miR-17 family, and three belong to the miR-15 family.

The miRNAs that were significantly associated with case status have critical roles in immune cell function. First, miR-17-5p, the most significantly downregulated miRNA, belongs to a cluster of miRNAs located in intron 3 of *C13orf25* at chromosome 13 [32]. This cluster of miRNAs (miR-17/92) has a wide range of functions in immune cell development and differentiation [32]. Specifically, the miR-17 cluster has been found to promote T cell survival, regulate Th1 response, and interleukin 10 (IL10) production in regulatory T cells [33,34]. Members of two other clusters (miR-106a/363 and miR-106b/25) of the miR-17 family were also significantly downregulated in cases. miR-106a on the X chromosome has been shown to downregulate IL10 expression [35], and all three miR-17 family members differentially expressed in our study (106a, 106b, 17-5p) are known to be upregulated in activated T lymphocytes [36]. Second, three members of the miR-15 family (miR-15a-5p, miR-15b-5p, miR-16-5p) were also significantly downregulated in cases. Expression of miR-15 has been found to enhance the induction of regulatory T cells from naïve CD4+ T cells [37]. Additionally, miR-210 suppresses proinflammatory cytokines in murine macrophages [38]. Finally, in considering experimentally verified targets of the differentially expressed miRNAs in this study, the *BCL2*, *CCND1*, and *VEGFA* genes are each targeted by five miRNAs from at least two miRNA families. These genes are known factors in apoptosis and cell survival, having previously been studied in the context of cancer pathways [39,40]. Taken together, the downregulation of the above miRNAs in cases suggests a lower immune activation state. Whether this lower activation state has implications for CKD progression or complications requires further investigation.

Population-based studies have reported increased risk of infection among persons with early stages of CKD. In older adults (age ≥ 65 years), reduced kidney function (eGFR < 60 mL/min/1.73m^2^) is associated with increased risk of infection-related hospitalization or bloodstream infection [8,9]. In persons with ESRD, immune dysfunction due to metabolic disorder and retention of uremic solute is well established [10,11]. The decline in kidney function occurs in a continuum. Thus, immune dysfunction may play a role in earlier stages of CKD. The increased risk of infection in persons with earlier stages of CKD is consistent with our findings of the downregulation of some miRNAs that are critical for immune cell function.

On kidney disease, the plasma levels of miR-15b were found to be 2-fold lower in hemodialysis patients versus non-CKD controls [41]. This result is consistent with our study. The associations between levels of miR-15b and miR-17 in kidney tissues and acute kidney injury in human and in animal models have been reported [42]. Since gene expression levels are highly tissue specific, it is uncertain whether studies of gene expression in one tissue may be generalizable to a different tissue [43–47].

### Strengths and limitations

One of the strengths of this study is its design. The cases and controls were carefully selected and matched for important potential confounders of gene expression, including hypertension medication use. In addition, we performed deep sequencing of the miRNA pool that allowed the discovery of novel miRNAs. However, our study has some limitations as well. First, the differentially expressed miRNAs need to be replicated in an independent study and validated by alternate laboratory methods, such as real-time polymerase chain reaction. Second, gene expression levels were measured in whole blood and not in specific types of immune cells. Thus, although cases and controls did not differ in major white blood cell types, and we used surrogate variables to control for unmeasured confounders, we cannot exclude the possibility that the differentially expressed miRNAs arise from differences in the distributions of white blood cell subpopulations. Third, our study is a cross-sectional study. We cannot distinguish whether the differential expression of miRNAs might have influenced disease development or was a consequence of the disease condition. Fourth, in contrast to miRNA co-expression network analysis, mRNA co-expression network analysis did not detect any significant modules although one module was nominally significant. This suggests larger sample size may be required for mRNA differential expression analysis. Finally, our cases and controls were under hypertension treatment, therefore our results cannot be generalized to persons without hypertension. Since the majority (>60%) of older adults in the U.S. are hypertensive [18], our results are, however, relevant to a large proportion of older adults.

## Supporting information

S1 Text Supplementary methods for mRNA and miRNA library preparation, sequencing, processing, alignment, and quantitation. (DOCX)

S1 Fig A heat map of the normalized count of miRNAs with nominal p-value < 0.01 in differential expression analysis. Hierarchical clustering was conducted using Euclidean distance and the Ward’s criterion [48].
(PDF)

S2 Fig Summary of mRNA and miRNA analyses and results in this study. (PDF)

S1 Table mRNA quality control and mapping statistics. Mapping statistics, Quality Control statistics from PicardTools modules, and ERCC statistics.
(XLSX)

S2 Table miRNA quality control and mapping statistics. Mapping and Quality Control filtering statistics.
(XLSX)

S3 Table Top genes from differential expression analysis using DESeq2 (p<1x10^-4^). (XLSX)

S4 Table Top transcripts from differential expression analysis using Ballgown (p<1x10^-3^). (XLSX)

S5 Table Association of miRNAs in the miR-17 and the miR-15 families with case status. (XLSX)

S6 Table Experimentally validated target genes for 11 differentially expressed miRNAs from miRTarBase. Three out of 14 differentially expressed miRNAs were not available in the database.
(XLSX)

S7 Table Association between experimentally validated target genes of the significant miRNA and case status. (XLSX)

S8 Table mRNA in each module from weighted gene co-expression network analysis. (XLSX)

S9 Table Association between the eigengene of each mRNA coexpression module and case status. The eigengene is the first principal component of expression levels of a module.
(XLSX)

S10 Table miRNAs in each correlated module from weighted gene co-expression network analysis. (XLSX)

S11 Table Top GO Terms for targets of differentially expressed miRNAs (one-tailed p<0.05). These terms surpass nominal significance, but do not reach significance after BH correction of p-values for number of terms.
(XLSX)

## References

[1] U.S. Renal Data System, USRDS 2012 Annual Data Report: Atlas of Chronic Kidney Disease and End-Stage Renal Disease in the United States, National Institutes of Health, National Institute of Diabetes and Digestive and Kidney Diseases, Bethesda, MD, 2012.

[2] Kopp JB. Rethinking hypertensive kidney disease: arterionephrosclerosis as a genetic, metabolic, and inflammatory disorder. Curr Opin Nephrol Hypertens. 2013 5;22(3):266–72. 23470819 10.1097/MNH.0b013e3283600f8c PMC4165431

[3] Harrison DG, Guzik TJ, Lob HE, Madhur MS, Marvar PJ, Thabet SR, et al Inflammation, immunity, and hypertension. Hypertens Dallas Tex 1979. 2011 2;57(2):132–40. 10.1161/HYPERTENSIONAHA.110.163576 PMC3028593 21149826

[4] Erlinger TP, Tarver-Carr ME, Powe NR, Appel LJ, Coresh J, Eberhardt MS, et al Leukocytosis, hypoalbuminemia, and the risk for chronic kidney disease in US adults. Am J Kidney Dis Off J Natl Kidney Found. 2003 8;42(2):256–63. 10.1016/s0272-6386(03)00650-4 12900806

[5] Bash LD, Erlinger TP, Coresh J, Marsh-Manzi J, Folsom AR, Astor BC. Inflammation, hemostasis, and the risk of kidney function decline in the Atherosclerosis Risk in Communities (ARIC) Study. Am J Kidney Dis Off J Natl Kidney Found. 2009 4;53(4):596–605. 10.1053/j.ajkd.2008.10.044 PMC2778194 19110358

[6] Sesso HD, Jiménez MC, Wang L, Ridker PM, Buring JE, Gaziano JM. Plasma Inflammatory Markers and the Risk of Developing Hypertension in Men. J Am Heart Assoc. 2015 9;4(9):e001802 doi: 10.1161/JAHA.115.001802 26391130 10.1161/JAHA.115.001802 PMC4599490

[7] Mattace-Raso FUS, Verwoert GC, Hofman A, Witteman JCM. Inflammation and incident-isolated systolic hypertension in older adults: the Rotterdam study. J Hypertens. 2010 5;28(5):892–5. doi: 10.1097/HJH.0b013e328336ed26 20375909 10.1097/HJH.0b013e328336ed26

[8] Dalrymple LS, Katz R, Kestenbaum B, de Boer IH, Fried L, Sarnak MJ, et al The risk of infection-related hospitalization with decreased kidney function. Am J Kidney Dis Off J Natl Kidney Found. 2012 3;59(3):356–63. 10.1053/j.ajkd.2011.07.012 PMC3288732 21906862

[9] James MT, Laupland KB, Tonelli M, Manns BJ, Culleton BF, Hemmelgarn BR, et al Risk of bloodstream infection in patients with chronic kidney disease not treated with dialysis. Arch Intern Med. 2008 11 24;168(21):2333–9. doi: 10.1001/archinte.168.21.2333 19029498 10.1001/archinte.168.21.2333

[10] Vanholder R, Ringoir S. Infectious morbidity and defects of phagocytic function in end-stage renal disease: a review. J Am Soc Nephrol JASN. 1993 3;3(9):1541–54. 8507809 10.1681/ASN.V391541

[11] Chonchol M. Neutrophil dysfunction and infection risk in end-stage renal disease. Semin Dial. 2006 8;19(4):291–6. doi: 10.1111/j.1525-139X.2006.00175.x 16893406 10.1111/j.1525-139X.2006.00175.x

[12] Zaza G, Granata S, Rascio F, Pontrelli P, Dell’Oglio MP, Cox SN, et al A specific immune transcriptomic profile discriminates chronic kidney disease patients in predialysis from hemodialyzed patients. BMC Med Genomics. 2013;6:17 doi: 10.1186/1755-8794-6-17 23663527 10.1186/1755-8794-6-17 PMC3655909

[13] Marketou ME, Kontaraki J, Zacharis E, Parthenakis F, Maragkoudakis S, Gavras I, et al Differential gene expression of bradykinin receptors 1 and 2 in peripheral monocytes from patients with essential hypertension. J Hum Hypertens. 2014 7;28(7):450–5. doi: 10.1038/jhh.2013.133 24401952 10.1038/jhh.2013.133

[14] Trionfini P, Benigni A, Remuzzi G. MicroRNAs in kidney physiology and disease. Nat Rev Nephrol. 2015 1;11(1):23–33. doi: 10.1038/nrneph.2014.202 25385286 10.1038/nrneph.2014.202

[15] Ichii O, Otsuka S, Sasaki N, Namiki Y, Hashimoto Y, Kon Y. Altered expression of microRNA miR-146a correlates with the development of chronic renal inflammation. Kidney Int. 2012 2;81(3):280–92. doi: 10.1038/ki.2011.345 21975861 10.1038/ki.2011.345

[16] Li S, Zhu J, Zhang W, Chen Y, Zhang K, Popescu LM, et al Signature microRNA expression profile of essential hypertension and its novel link to human cytomegalovirus infection. Circulation. 2011 7 12;124(2):175–84. doi: 10.1161/CIRCULATIONAHA.110.012237 21690488 10.1161/CIRCULATIONAHA.110.012237

[17] The Atherosclerosis Risk in Communities (ARIC) Study: design and objectives. The ARIC investigators. Am J Epidemiol. 1989 4;129(4):687–702. 2646917

[18] Egan BM, Zhao Y, Axon RN. US trends in prevalence, awareness, treatment, and control of hypertension, 1988–2008. JAMA. 2010 5 26;303(20):2043–50. doi: 10.1001/jama.2010.650 20501926 10.1001/jama.2010.650

[19] Napoleone E, Di Santo A, Camera M, Tremoli E, Lorenzet R. Angiotensin-converting enzyme inhibitors downregulate tissue factor synthesis in monocytes. Circ Res. 2000 2 4;86(2):139–43. 10666408 10.1161/01.res.86.2.139

[20] Parsa A, Kao WHL, Xie D, Astor BC, Li M, Hsu C, et al APOL1 risk variants, race, and progression of chronic kidney disease. N Engl J Med. 2013 12 5;369(23):2183–96. doi: 10.1056/NEJMoa1310345 24206458 10.1056/NEJMoa1310345 PMC3969022

[21] Leek JT. svaseq: removing batch effects and other unwanted noise from sequencing data. Nucleic Acids Res. 2014 12 1;42(21). 10.1093/nar/gku864 PMC4245966 25294822

[22] Benjamini Y, Hochberg Y. Controlling the False Discovery Rate: A Practical and Powerful Approach to Multiple Testing. J R Stat Soc Ser B Methodol. 1995;57(1):289–300.

[23] Love MI, Huber W, Anders S. Moderated estimation of fold change and dispersion for RNA-seq data with DESeq2. Genome Biol. 2014;15(12):550 doi: 10.1186/s13059-014-0550-8 25516281 10.1186/s13059-014-0550-8 PMC4302049

[24] Uhlén M, Fagerberg L, Hallström BM, Lindskog C, Oksvold P, Mardinoglu A, et al Proteomics. Tissue-based map of the human proteome. Science. 2015 1 23;347(6220):1260419 doi: 10.1126/science.1260419 25613900 10.1126/science.1260419

[25] Frazee AC, Pertea G, Jaffe AE, Langmead B, Salzberg SL, Leek JT. Ballgown bridges the gap between transcriptome assembly and expression analysis. Nat Biotechnol. 2015 3;33(3):243–6. doi: 10.1038/nbt.3172 25748911 10.1038/nbt.3172 PMC4792117

[26] Kozomara A, Griffiths-Jones S. miRBase: annotating high confidence microRNAs using deep sequencing data. Nucleic Acids Res. 2014 1;42(Database issue):D68–73. doi: 10.1093/nar/gkt1181 24275495 10.1093/nar/gkt1181 PMC3965103

[27] Langfelder P, Horvath S. WGCNA: an R package for weighted correlation network analysis. BMC Bioinformatics. 2008;9:559 doi: 10.1186/1471-2105-9-559 19114008 10.1186/1471-2105-9-559 PMC2631488

[28] Bleazard T, Lamb JA, Griffiths-Jones S. Bias in microRNA functional enrichment analysis. Bioinforma Oxf Engl. 2015 5 15;31(10):1592–8. 10.1093/bioinformatics/btv023 PMC4426843 25609791

[29] Enright AJ, John B, Gaul U, Tuschl T, Sander C, Marks DS. MicroRNA targets in Drosophila. Genome Biol. 2003;5(1):R1 doi: 10.1186/gb-2003-5-1-r1 14709173 10.1186/gb-2003-5-1-r1 PMC395733

[30] Kinsella RJ, Kähäri A, Haider S, Zamora J, Proctor G, Spudich G, et al Ensembl BioMarts: a hub for data retrieval across taxonomic space. Database J Biol Databases Curation. 2011;2011:bar030. 10.1093/database/bar030 PMC3170168 21785142

[31] Chou C-H, Chang N-W, Shrestha S, Hsu S-D, Lin Y-L, Lee W-H, et al miRTarBase 2016: updates to the experimentally validated miRNA-target interactions database. Nucleic Acids Res. 2016 1 4;44(D1):D239–47. doi: 10.1093/nar/gkv1258 26590260 10.1093/nar/gkv1258 PMC4702890

[32] Mogilyansky E, Rigoutsos I. The miR-17/92 cluster: a comprehensive update on its genomics, genetics, functions and increasingly important and numerous roles in health and disease. Cell Death Differ. 2013 12;20(12):1603–14. doi: 10.1038/cdd.2013.125 24212931 10.1038/cdd.2013.125 PMC3824591

[33] Jiang S, Li C, Olive V, Lykken E, Feng F, Sevilla J, et al Molecular dissection of the miR-17-92 cluster’s critical dual roles in promoting Th1 responses and preventing inducible Treg differentiation. Blood. 2011 11 17;118(20):5487–97. doi: 10.1182/blood-2011-05-355644 21972292 10.1182/blood-2011-05-355644 PMC3217351

[34] de Kouchkovsky D, Esensten JH, Rosenthal WL, Morar MM, Bluestone JA, Jeker LT. microRNA-17-92 regulates IL-10 production by regulatory T cells and control of experimental autoimmune encephalomyelitis. J Immunol Baltim Md 1950. 2013 8 15;191(4):1594–605. 10.4049/jimmunol.1203567 PMC4160833 23858035

[35] Sharma A, Kumar M, Aich J, Hariharan M, Brahmachari SK, Agrawal A, et al Posttranscriptional regulation of interleukin-10 expression by hsa-miR-106a. Proc Natl Acad Sci U S A. 2009 4 7;106(14):5761–6. doi: 10.1073/pnas.0808743106 19307576 10.1073/pnas.0808743106 PMC2659714

[36] Grigoryev YA, Kurian SM, Hart T, Nakorchevsky AA, Chen C, Campbell D, et al MicroRNA regulation of molecular networks mapped by global microRNA, mRNA, and protein expression in activated T lymphocytes. J Immunol Baltim Md 1950. 2011 9 1;187(5):2233–43. 10.4049/jimmunol.1101233 PMC3159804 21788445

[37] Singh Y, Garden OA, Lang F, Cobb BS. MicroRNA-15b/16 Enhances the Induction of Regulatory T Cells by Regulating the Expression of Rictor and mTOR. J Immunol Baltim Md 1950. 2015 12 15;195(12):5667–77. 10.4049/jimmunol.1401875 PMC4671309 26538392

[38] Qi J, Qiao Y, Wang P, Li S, Zhao W, Gao C. microRNA-210 negatively regulates LPS-induced production of proinflammatory cytokines by targeting NF-κB1 in murine macrophages. FEBS Lett. 2012 4 24;586(8):1201–7. doi: 10.1016/j.febslet.2012.03.011 22575656 10.1016/j.febslet.2012.03.011

[39] Tang S-W, Chang W-H, Su Y-C, Chen Y-C, Lai Y-H, Wu P-T, et al MYC pathway is activated in clear cell renal cell carcinoma and essential for proliferation of clear cell renal cell carcinoma cells. Cancer Lett. 2009 1 8;273(1):35–43. doi: 10.1016/j.canlet.2008.07.038 18809243 10.1016/j.canlet.2008.07.038

[40] Wang H, Xu Z, Ma M, Wang N, Wang K. Network analysis of microRNAs, transcription factors, target genes and host genes in nasopharyngeal carcinoma. Oncol Lett. 2016 6;11(6):3821–8. doi: 10.3892/ol.2016.4476 27313701 10.3892/ol.2016.4476 PMC4888083

[41] Wang H, Peng W, Ouyang X, Dai Y. Reduced circulating miR-15b is correlated with phosphate metabolism in patients with end-stage renal disease on maintenance hemodialysis. Ren Fail. 2012;34(6):685–90. doi: 10.3109/0886022X.2012.676491 22512691 10.3109/0886022X.2012.676491

[42] Fan P-C, Chen C-C, Chen Y-C, Chang Y-S, Chu P-H. MicroRNAs in acute kidney injury. Hum Genomics. 2016 9 8;10(1):29 doi: 10.1186/s40246-016-0085-z 27608623 10.1186/s40246-016-0085-z PMC5016954

[43] Rodwell GEJ, Sonu R, Zahn JM, Lund J, Wilhelmy J, Wang L, et al A transcriptional profile of aging in the human kidney. PLoS Biol. 2004 12;2(12):e427 doi: 10.1371/journal.pbio.0020427 15562319 10.1371/journal.pbio.0020427 PMC532391

[44] Fu J, Wolfs MGM, Deelen P, Westra H-J, Fehrmann RSN, Te Meerman GJ, et al Unraveling the regulatory mechanisms underlying tissue-dependent genetic variation of gene expression. PLoS Genet. 2012 1;8(1):e1002431 doi: 10.1371/journal.pgen.1002431 22275870 10.1371/journal.pgen.1002431 PMC3261927

[45] GTEx Consortium. Human genomics. The Genotype-Tissue Expression (GTEx) pilot analysis: multitissue gene regulation in humans. Science. 2015 5 8;348(6235):648–60. doi: 10.1126/science.1262110 25954001 10.1126/science.1262110 PMC4547484

[46] Ludwig N, Leidinger P, Becker K, Backes C, Fehlmann T, Pallasch C, et al Distribution of miRNA expression across human tissues. Nucleic Acids Res. 2016 5 5;44(8):3865–77. doi: 10.1093/nar/gkw116 26921406 10.1093/nar/gkw116 PMC4856985

[47] Fehlmann T, Ludwig N, Backes C, Meese E, Keller A. Distribution of microRNA biomarker candidates in solid tissues and body fluids. RNA Biol. 2016 11;13(11):1084–8. doi: 10.1080/15476286.2016.1234658 27687236 10.1080/15476286.2016.1234658 PMC5100342

[48] Murtagh F. & Legendre P. J Classif (2014) 31: 274 doi: 10.1007/s00357-014-9161-z

