# Supplementary material for: MicroRNAs in the miR-17 and miR-15 families are downregulated in chronic kidney disease with hypertension
Source: PLoS One. 2017 Aug 3;12(8):e0176734. doi: 10.1371/journal.pone.0176734 (PMC5542606; doi:10.1371/journal.pone.0176734)
Supplement: S1 Text — (DOCX) [file pone.0176734.s001.docx]

**Supplementary Methods for mRNA and miRNA library preparation, sequencing, processing, alignment, and quantitation**

*Total RNA isolation*

Total RNA was isolated in the Human Genetics Center (HGC) Laboratory at the University of Texas Health Science Center at Houston School of Public Health from PAXgene preserved whole blood of ARIC study participants using the MagMAX for Stabilized Blood Tubes RNA Isolation Kit (ThermoFisher Scientific, Waltham, MA). RNA integrity was assessed with Agilent RNA 6000 Nano Kits using the Agilent 2100 Bioanalyzer (Agilent Technologies; Santa Clara, CA, USA).

*Globin depletion, mRNA library preparation and sequencing*

After RNA isolation, the HGC Laboratory used the GLOBINclear Kit (ThermoFisher Scientific) for the purpose of reducing >95% of the alpha and beta globin mRNA. Globin depleted samples were then provided to the Baylor College of Medicine Human Genome Sequencing Center (BCM-HGSC) for library preparation and sequencing of mRNA.. Following a custom protocol, polyadenylated mRNA was isolated from 1μg of total RNA (RIN≥7) from PAXgene-preserved whole blood via poly-A pulldown with Oligo(dT)25 Dynabeads, and fragmented using heat. The Superscript III reverse transcriptase was used for first strand cDNA synthesis, followed by the inclusion of dUTP during second strand synthesis for stranded library preparation. Subsequently, end-repair and 3’ adenylation of cDNA were performed to prepare for Illumina adapter ligation for paired-end libraries, after which the second strand (non-coding) was digested with the Uracil-DNA Glycosylase enzyme.

Exogenous RNAs from the External RNA Controls Consortium (ERCC) [1] were added at the beginning of library preparation to total RNA. This set of 92 polyadenylated transcripts was incorporated to track sample fluctuations. Sequencing was carried out on the Illumina HiSeq 2000 with five libraries pooled per lane producing ~35M paired-end reads of 101 base pairs (bp) per sample.

*miRNA library preparation and sequencing*

Total RNA isolated from PAXgene-preserved whole blood as described above were also used for library preparation and miRNA sequencing at the BCM-HGSC using the Illumina^®^ TruSeq^®^ small RNA sample preparation kit, beginning with 200 ng to 1μg of total RNA. First, the Illumina 3’ adapter, specifically targeting small RNAs, was ligated, followed by the Illumina 5’ adapter. The Superscript II reverse transcriptase (Life Technologies, cat# 18064-014) was used to synthesize single-stranded cDNA, which was amplified by PCR and quantified on the Agilent Bioanalyzer 2100. A maximum of 10 barcoded libraries were pooled in equimolar ratios and loaded on 6% PAGE gels, and subsequently the 145-160bp region representing miRNAs was extracted. Sequencing of pooled libraries was done on the Illumina HiSeq 2500, to produce ~8M single-end 36 bp reads per sample.

*mRNA read processing, mapping and quantitation*

The raw mRNA data were available as FASTQ files. The Cutadapt [2] Python script (v1.8.1) was used to trim reads with at least five base overlap with Illumina adapters, terminal polyA or polyT stretches of length 3-12 nucleotides (nt) [3] with error rate of 10%, and terminal uncalled bases, retaining those only of minimum length 24 with no more than 10% uncalled bases.

Annotation-assisted alignment was performed with STAR [4] v2.4.2a to the GRCh37 reference genome, allowing a maximum mismatch fraction relating to read length of 0.035. The reference FASTA files consisted of the main chromosomes, patches and scaffolds from GRCh37 “Lite” version (available from ftp://ftp.ncbi.nih.gov/genbank/genomes/Eukaryotes/vertebrates_mammals/Homo_sapiens/GRCh37/special_requests/ GRCH37-lite.fa.gz), along with the 92 ERCC sequences (available from

 http://tools.invitrogen.com/downloads/ERCC92.fa). Annotations were prepared from GENCODE V19 (<http://www.gencodegenes.org/releases/19.html>) with the comprehensive gene annotation file (gencode.v19.chr_patch_hapl_scaff.annotation.gtf), the tRNA file (gencode.v19.tRNAs.gtf), and ERCC annotations. The PicardTools [5] (v1.136) CollectRnaSeqMetrics module, featureCounts [6] from the subread v1.4.6 package, and R [7] were used to assess quality control (QC), ERCC, and alignment metrics.

To prepare for analysis of features at both the gene and transcript levels, quantitation was done for both types of features. The featureCounts program was used to count fragments, including unique reads only, for gene-level analysis. For transcript-level analysis, the StringTie [8] v1.2.2 program was used for reference-guided transcript assembly.

*miRNA read processing, mapping and quantitation*

The raw miRNA data were available as FASTQ files. The Cutadapt Python script (v1.8.1) and miRDeep2 [9] (v0.0.7) software package were used to process, map and quantitate miRNA expression levels. Cutadapt was used to trim reads with minimum four base overlap with Illumina adapter sequences (primarily the 3’ adapter), Phred-scaled quality score cutoff of 20 at both the 3’ and 5’ ends, terminal uncalled bases, and those with high homopolymer content, retaining those with minimum length 15 and a maximum of 5% uncalled bases. The reads were further processed and collapsed by mirRDeep2’s mapper.pl script, keeping only those minimum 18 bases in length and supported by at least four reads. The miRDeep2 package enlists the software bowtie [10] (v1.1.2) for alignment with default parameters, using the GRCh37 reference genome as described for mRNA analyses, and human mature miRNAs and precursors from miRBase [11] v20 were used as reference sequences.

In the first step of a two-pass scheme recommended by the miRDeep2 authors (S. Mackowiak, written communication, April 2016), novel miRNA identification and quantitation of known and novel miRNAs were performed with the miRDeep2.pl script, which calls the quantifier.pl module for the known miRNA quantitation. Subsequently, novel miRNAs with miRDeep2 score ≥4, corresponding to a signal-to-noise ratio >10 in a minimum of five out of the 30 samples were carried forward and appended to the reference mature miRNA FASTA file. The quantifier.pl module was then used to re-quantitate annotated miRNAs, including these novel miRNAs as described. As miRDeep2 estimates precursor source for each mature miRNA, multi-mapped reads were divided among precursors of mature miRNAs.

**References**

1. Lemire A, Lea K, Batten D, Jian Gu S, Whitley P, Bramlett K, et al. Development of ERCC RNA Spike-In Control Mixes. J Biomol Tech JBT. 2011 Oct;22(Suppl):S46.

2. Martin M. Cutadapt removes adapter sequences from high-throughput sequencing reads. EMBnet.journal. 2011 May 2;17(1):10.

3. Gupta S, Ellis SE, Ashar FN, Moes A, Bader JS, Zhan J, et al. Transcriptome analysis reveals dysregulation of innate immune response genes and neuronal activity-dependent genes in autism. Nat Commun. 2014;5:5748.

4. Dobin A, Davis CA, Schlesinger F, Drenkow J, Zaleski C, Jha S, et al. STAR: ultrafast universal RNA-seq aligner. Bioinforma Oxf Engl. 2013 Jan 1;29(1):15–21.

5. Picard Tools webpage. [ http://picard.sourceforge.net].

6. Liao Y, Smyth GK, Shi W. featureCounts: an efficient general purpose program for assigning sequence reads to genomic features. Bioinforma Oxf Engl. 2014 Apr 1;30(7):923–30.

7. R Core Team (2016). R: A language and environment for statistical computing. R Foundation for Statistical Computing, Vienna, Austria. URL https://www.R-project.org/.

8. Pertea M, Pertea GM, Antonescu CM, Chang T-C, Mendell JT, Salzberg SL. StringTie enables improved reconstruction of a transcriptome from RNA-seq reads. Nat Biotechnol. 2015 Mar;33(3):290–5.

9. Friedländer MR, Mackowiak SD, Li N, Chen W, Rajewsky N. miRDeep2 accurately identifies known and hundreds of novel microRNA genes in seven animal clades. Nucleic Acids Res. 2012 Jan;40(1):37–52.

10. Langmead B, Trapnell C, Pop M, Salzberg SL. Ultrafast and memory-efficient alignment of short DNA sequences to the human genome. Genome Biol. 2009;10(3):R25.

11. Kozomara A, Griffiths-Jones S. miRBase: annotating high confidence microRNAs using deep sequencing data. Nucleic Acids Res. 2014 Jan;42(Database issue):D68–73.
