# Supplementary material for: MicroRNAs in the miR-17 and miR-15 families are downregulated in chronic kidney disease with hypertension
Source: PLoS One. 2017 Aug 3;12(8):e0176734. doi: 10.1371/journal.pone.0176734 (PMC5542606; doi:10.1371/journal.pone.0176734)
Supplement: S1 Fig — Hierarchical clustering was conducted using Euclidean distance and the Ward’s criterion [48]. (PDF) [file pone.0176734.s002.pdf]

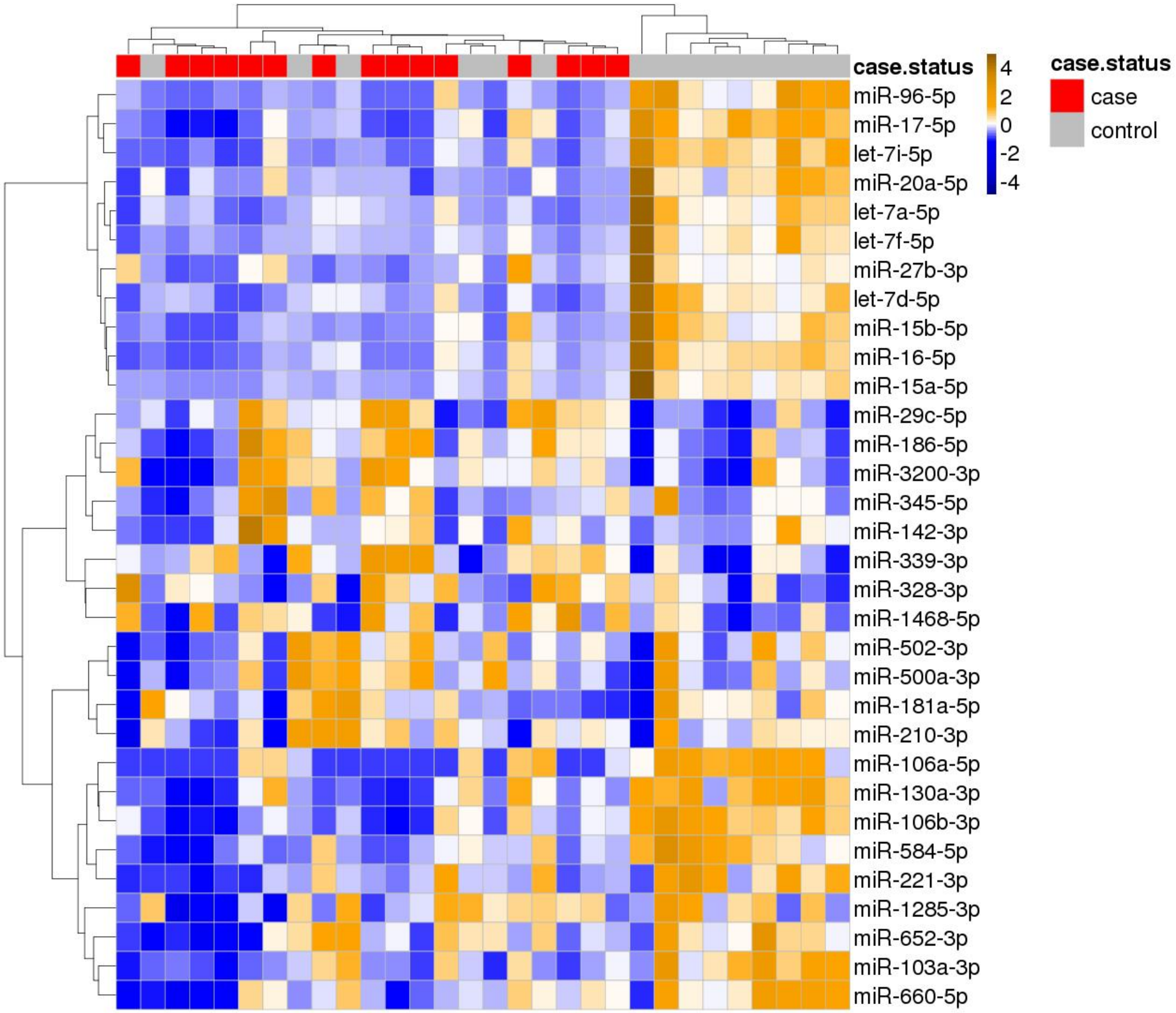

Figure S1. A heat map of the normalized count of miRNAs with nominal p-value < 0.01 in differential expression analysis. Hierarchical clustering was conducted using Euclidean distance and the Ward's criterion. (Murtagh et al. (2014). Ward's hierarchical agglomerative clustering method: which algorithms implement Ward's criterion? Journal of Classification)
