# Supplementary figures and images for: MicroRNAs in the miR-17 and miR-15 families are downregulated in chronic kidney disease with hypertension
Source: PLoS One. 2017 Aug 3;12(8):e0176734. doi: 10.1371/journal.pone.0176734 (PMC5542606; doi:10.1371/journal.pone.0176734)

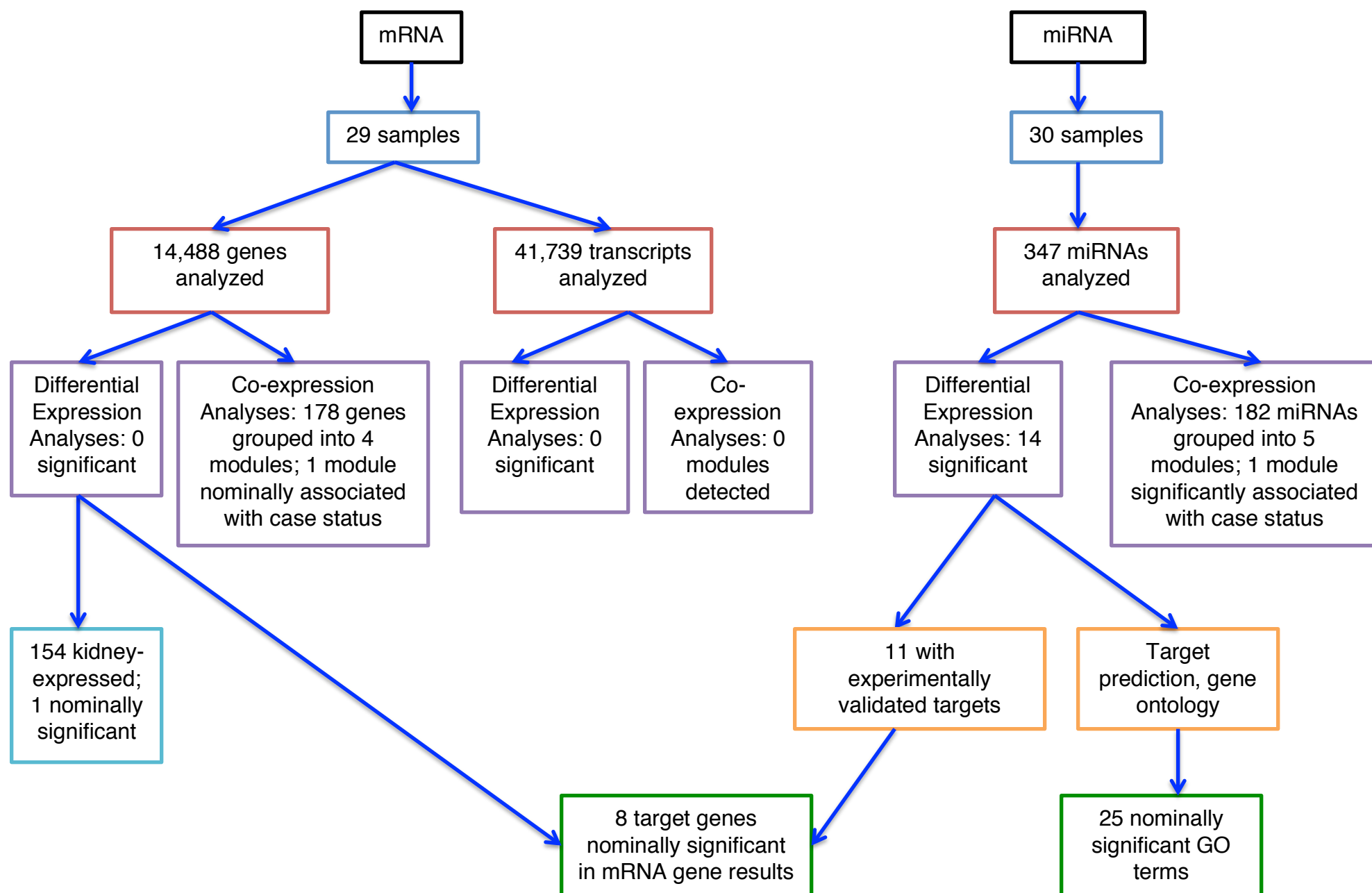

Figure S2. Summary of mRNA and miRNA analyses and results in this study

Supplement: S2 Fig — (PDF) [file pone.0176734.s003.pdf]
